# Molecular characterization of multidrug resistant *Acinetobacter baumannii* clinical isolates from Alexandria, Egypt

**DOI:** 10.3389/fcimb.2023.1208046

**Published:** 2023-07-20

**Authors:** Sandra Sánchez-Urtaza, Alain Ocampo-Sosa, Ainhoa Molins-Bengoetxea, Mohammed A. El-Kholy, Marta Hernandez, David Abad, Sherine M. Shawky, Itziar Alkorta, Lucia Gallego

**Affiliations:** ^1^ Laboratory of Antibiotics and Molecular Bacteriology, Department of Immunology, Microbiology and Parasitology, Faculty of Medicine and Nursing, University of the Basque Country, Leioa, Spain; ^2^ Microbiology Service, University Hospital Marqués de Valdecilla, Health Research Institute (Instituto de Investigación Valdecilla), Santander, Spain and CIBERINFEC, Instituto de Salud Carlos III, Madrid, Spain; ^3^ Department of Microbiology and Biotechnology, Division of Clinical and Biological Sciences, College of Pharmacy, Arab Academy for Science, Technology & Maritime Transport (AASTMT), Alexandria, Egypt; ^4^ Laboratory of Molecular Biology and Microbiology, One Health, Agricultural Technological, Institute of Castile and Leon (ITACyL), Valladolid, Spain; ^5^ Medical Research Institute, Alexandria University, Alexandria, Egypt; ^6^ Department of Biochemistry and Molecular Biology, Faculty of Science and Technology, University of the Basque Country, Leioa, Spain

**Keywords:** *Acinetobacter baumannii*, carbapenemase genes, antibiotic resistance, molecular epidemiology, whole genome sequencing

## Abstract

Carbapenem resistant *Acinetobacter baumannii* is a major global concern, especially in countries of the Middle East and North Africa, where the antibiotic resistance rates are on the rise. The aim of this study was to study the genomic characteristics and antimicrobial susceptibility profile of thirty-six multidrug resistant *A. baumannii* clinical isolates obtained in hospitals from Alexandria, Egypt. Antibiotic resistance rates were estimated by determination of Minimum Inhibitory Concentrations. Carbapenemase genes, other antibiotic resistance genes and virulence factors were then screened by the use of Whole Genome Sequencing. Isolates were also subjected to Multi Locus Sequence Typing (MLST) using the Pasteur Scheme and to core genome MLST to study their clonal relatedness. In addition, plasmid analysis was performed by the use of a commercial kit and S1- Pulsed Field Gel Electrophoresis, and Hybridization experiments with DIG-labeled DNA probes for *bla*_NDM-1_, *bl*a_PER-7_ and *bla*_GES-like_ were performed to locate these genes. The majority of isolates were resistant to β-lactams (including carbapenems), fluoroquinolones, aminoglycosides and trimethoprim; and some showed resistance to cefiderocol and minocycline. We identified 8 different *bla*_OXA-51-like_ variants including *bla*_OXA-51_, *bla*_OXA-64_, *bla*_OXA-65_, *bla*_OXA-66_, *bla*_OXA-68_, *bla*_OXA-91_, *bla*_OXA-94_ and *bla*_OXA-336_; *bla*_OXA-23_, *bla*_NDM-1_, *bla*_PER-7_, *bla*_GES-like_ and *bla*_ADC-like_ and other antibiotic resistance genes, some of these genes were within transposons or class 1 integrons. Multiple virulence factors responsible for adherence, biofilm production, type II and type VI secretion systems, exotoxins, exoenzymes, immune modulation and iron uptake were observed and 34 out of 36 isolates showed motility. Thirty-five out of 36 isolates clustered with International Clones 2, 4, 5, 7, 8 and 9; and 9 STs were identified including ST570, ST2, ST600, ST15, ST113, ST613, ST85, ST158, ST164. Plasmids ranging in size from 1.7 to 70 kb were found; *bla*_NDM-1_ and *bl*a_PER-7_ genes were located in the chromosome and *bla*_GES-like_ genes were simultaneously located in the chromosome and in a plasmid of 70kb. In conclusion, this study revealed a wide spectrum of antibiotic resistance genes and a variety of lineages among *A. baumannii* isolated in hospitals from Alexandria, and highlights the importance of investigating the molecular epidemiology to control the spread of multi-drug resistant isolates.

## 1 Introduction

Carbapenem resistant *Acinetobacter baumannii* (CRAB) is one of the major global threats for healthcare settings worldwide, as there are only few antibiotics effective to treat the infections caused by these isolates due to its high rates of antimicrobial resistance (Ejaz et al., 2021). This pathogen is responsible for multiple nosocomial infections such as bloodstream infections, urinary tract infections, wound infections, ventilator-associated pneumonia and other respiratory tract infections, meningitis and bacteremia (Kurihara et al., 2020). As a consequence, *A. baumannii* is heading the World Head Organization’s (WHO) list of critical pathogens for which new antibiotics are urgently needed (El-Kholy et al., 2021). Many other global institutions such as the European Centre for Disease Prevention and Control (ECDC), Infectious Diseases Society of America (IDSA) and the Center for Disease Control and Prevention of America (CDC) have also declared it an urgent threat (Mea et al., 2021).

Rates of mortality and disability caused by *A. baumannii* infections are increasing. Retrospective studies showed that the mortality rates associated with *A. baumannii* infections are ranging from 22.8% to 49.6% in the United States (US), and from 29% to 71.6% in Europe (Patel et al., 2019). According to autores, mortality associated with *A. baumannii* causing hospital-acquired and ventilator-associated pneumonia was higher in Western Asia (56.2%), Southern Europe (55.7%) and Northern Africa (53.3%). Countries of the Mediterranean area, such as Greece (68.2%), Turkey (61.4) or Egypt (53.3%) were the ones with the highest reported mortality rates (Mohd Sazlly Lim et al., 2019). Indeed, in Egypt 30–100% of *A. baumannii* isolates are considered as Multidrug-Resistant (MDR), and carbapenem resistance was reported in 26.6–100% of *A. baumannii* isolates (El-Kholy et al., 2021). Furthermore, many reports showed that the COVID-19 pandemic increased CRAB infection rates, for instance in the US, the rates in hospitals increased a 78% and overall by 35% in 2020 compared with 2019 (Centers for Disease Control and Prevention, 2022). In Egypt, MDR *A. baumannii* was the second most common cause of infection (27.4%) among mechanically ventilated patients during the second wave of COVID-19 (Elwakil et al., 2023). The economic impact is also remarkable in CRAB infections, for instance in the United States, the CDC reported that treatment costs were around $281 million in 2019 (Ejaz et al., 2021).

Different resistance mechanisms are commonly found in *A. baumannii* including antibiotic inactivation enzymes, alteration of target sites, overexpression of efflux pumps and loss of porins (Chakravarty, 2020). Carbapenemases are the main carbapenem resistance mechanism in *A. baumannii*, being carbapenem-hydrolyzing oxacilinases the most important ones. However, during the last years, class B metallo-β-lactamases such as New Delhi Metallo-Beta-lactamases (NDM) are on the rise (Xanthopoulou et al., 2020).

Nine major International Clones (IC1-9) of *A. baumannii*, have been described up to now, being IC2/CC92 with the acquired *bla*_OXA-23_ gene the most disseminated lineage worldwide (Al-Hassan et al., 2019; Al-Hassan et al., 2021); and IC1 and IC2 in Europe (Muthuirulandi Sethuvel et al., 2019). In the Middle East and North Africa, *A. baumannii* clinical outbreaks, caused by MDR isolates endemically producing carbapenemases NDM-1, NDM-2 and OXA-23, are usually poly-clonal but with dominance of IC2 lineage (Hassan et al., 2021). Although IC2 is the most disseminated clone in Northern Africa, isolates belonging to IC5 and IC9 are also reported (Al-Hassan et al., 2021; Hamed et al., 2022). From 2013 to date, isolates belonging to Oxford ST208 (IC2), are commonly reported in Egyptian hospitals (Al-Hassan et al., 2019). However, in spite of the alarming situation, there is little information about *A. baumannii* in Egypt and more studies are needed (Hassan et al., 2021). Nevertheless, the limited resources for research in low- and middle-income countries such as Egypt, makes it difficult to obtain epidemiological data. It is of high concern to investigate the molecular epidemiology to control the dissemination of these clinically important isolates.

The aim of the present study was to characterize the genetic features, to study the molecular epidemiology and to identify the antimicrobial susceptibility profiles of thirty-six carbapenem resistant *A. baumannii* clinical isolates obtained from hospitals from Alexandria, Egypt.

## 2 Materials and methods

### 2.1 Bacterial isolates, species identification and antimicrobial susceptibility testing

Thirty-six *A. baumannii* isolates were collected from August 2020 to February 2021 in hospitals from Alexandria, Egypt and processed and identified in the Medical Research Institute of Alexandria. The bacterial isolates were from 21 male and 15 female patients. Clinical samples were obtained from the following sources: bronchoalveolar lavage (14), swab (8), blood (5), aspirate (3), sputum (3), endotracheal tube (1), urine (1) and tissue (1).

Species identification was assessed by VITEK 2^®^ automated system (Biomérieux, Marcy-l’Étoile, France) and *gyrB* multiplex PCR (Higgins et al., 2010b). Minimum Inhibitory Concentrations (MICs) to ticarcillin, ticarcillin/clavulanic acid, piperacillin, piperacillin/tazobactam, ceftazidime, cefepime, amikacin, gentamicin, tobramycin, minocycline, ciprofloxacin, trimethoprim/sulfamethoxazole, imipenem, meropenem and colistin were determined by the use of VITEK 2^®^ automated system. Antimicrobial activity of cefiderocol was determined by disk diffusion method using 30 µg cefiderocol discs (ThermoFisher Scientific, Waltham, United States) following the EUCAST guidelines and clinical breakpoints (Versions 10.0 and 12.0, January 2022). *Escherichia coli* ATCC 25922 and *Pseudomonas aeruginosa* ATCC 27853 were used as control strains.

### 2.2 Detection of carbapenemase genes

Carbapenemase-encoding genes were analyzed by multiplex PCR including primers for: *bla*_OXA-23-like, −40-like, −51-like, −58-like, −143-like_, _and −235-like_ (Woodford et al., 2006; Higgins et al., 2010a; Higgins et al., 2013). Two additional multiplex PCR were performed to investigate the presence of *bla*_VIM_, *bla*_KPC_, *bla*_NDM_, *bla*_OXA-48_, *bla*_IMI_, *bla*_GES_, *bla*_GIM_, *bla*_IMP_ and ISAba-1/*bla*_OXA-51-like_ (Cerezales et al., 2021).

### 2.3 Whole Genome Sequencing, genome annotation, analysis and visualization and virulence factors analysis

Total DNA was purified with the DNeasy Blood and Tissue Kit (Qiagen, Hilden, Germany) and sequenced on a MiSeq device using reagents kit v3 for 2×300 paired-end libraries (Illumina) as previously described (Hernández et al., 2017). Raw reads from the sequencing platform were directly analyzed using the in-house bioinformatics pipeline TORMES^®^ (Quijada et al., 2019). *A. baumannii* ATCC 17978 was used as reference strain. The options used in this study included quality control and filtering of the reads by using Trimmomatic (Bolger et al., 2014), Prinseq (Schmieder and Edwards, 2011) and Kraken (Wood and Salzberg, 2014). Genome assembly was performed with SPAdes (Bankevich et al., 2012) and Quast (Gurevich et al., 2013) and genome annotation with Prokka software tool (Seemann, 2014). The whole-genome shotgun sequences of the isolates generated for this study were deposited and can be found in GenBank under the BioProject accession number PRJNA856145 and the accession numbers of each isolate are detailed in **Supplementary Table 1**. Search of antibiotic resistance genes was done using BLAST (Camacho et al., 2009) and ABRicate (https://github.com/tseemann/abricate (accessed on October 2021)) against ResFinder database (Zankari et al., 2012). Genome was edited and visualized by the use of SnapGene Viewer 6.0.5. Virulence factors were screened using Virulence Factors Database (VFDB) search tool (Liu et al., 2019) and Ridom SeqSphere+ software version 8.5.1 (Ridom GmbH,Münster, Germany).

### 2.4 Surface-associated motility

Motility assay was performed on Motility Test Medium (Condalab, Madrid, Spain) inoculated on the surface and incubated overnight at 37°C following manufacturer instructions.

### 2.5 Biofilm formation assays

Biofilm production was evaluated using the crystal violet staining assay described by O’Toole and Kolter as described before (O'Toole and Kolter, 1998) with slight modifications. Briefly, *A. baumannii* overnight cultures were adjusted to a 0.5 McFarland turbidity in 0.85% saline solution. Biofilms were developed in 24-well flat-bottom plates (Sarstedt^®^, Nümbrecht, Germany). First, bacterial suspensions were incubated at 37°C for 24 h. Then, biofilms were washed, air-dried and stained with 1 mL/well of 0.7% crystal violet solution (Sigma-Aldrich). Finally, stained biofilms were solubilized with 1mL/well of 33% acetic acid solution (Sigma-Aldrich).Biofilm production was determined at 600 nm using the Tecan Infinite M200 Pro Microplate Reader (Tecan Group Ltd., Männedorf, Suiza). Results were corrected for background staining by subtracting the value for crystal violet bound to uninoculated Müller Hinton Broth control wells. Isolates *E. coli* J53 and *P. aeruginosa* PAO1 were used as negative and positive controls, respectively. The experiments were performed in triplicate and repeated in three different days with similar results.

### 2.6 Molecular typing

Multi-Locus Sequence Typing (MLST) was performed using an open-source tool (MLST, T. Seemann, https://github.com/tseemann/mlst (accessed on October 2021) following Pasteur typing scheme. The *bla*_OXA-51-like_ variant combined with the Sequence Type (ST) were used to assign the isolate to an International Clone (IC). Core genome MLST (cgMLST) based on a core genome of 2390 alleles was also carried out to study clonal relatedness by the use of Ridom SeqSphere+ software version 8.5.1 (Ridom GmbH) and a minimum spanning tree was generated.

### 2.7 Plasmid analysis and carbapenemase genes localization

Plasmid extractions were carried out by the use of GeneJET Plasmid Miniprep Kit following manufacturer indications (ThermoFisher Scientific, Waltham, Massachusetts, USA) and S1-Pulsed-Field Gel Electrophoresis (PFGE). Bacterial DNA embedded in agarose plugs was digested using 14 units of S1-nuclease (Takara Bio, Kusatsu, Japan) per plug followed by PFGE. Samples were run on a CHEF-DR III system (Bio-Rad, Munich, Germany) for 20 h at 6 V/cm and 14°C. CHEF DNA Size Standard Lambda Ladder (Bio-Rad) was used as molecular weight marker. Southern blot hybridizations were performed to locate *bla*_NDM-1_, *bla*_PER-7_ and *bla*_GES-like_ genes with specific digoxigenin-labeled DNA probes (Roche, Mannheim, Germany). Signal detection was performed using DIG Nucleic Acid Detection Kit (Roche). Determination of the presence and classification of replicase genes was conducted using *A. baumannii* PCR-Based Replicon Typing as previously described (Bertini et al., 2010).

## 3 Results

### 3.1 Bacterial isolates, species identification and antimicrobial susceptibility testing

The thirty-six bacterial isolates were identified by VITEK 2^®^ and *gyrB* multiplex PCR as *A. baumannii* (**Supplementary Figure 1**). Fifty-eight percent of the isolates were from male patients vs. forty-two percent from female patients. Data regarding to collection date, sex and type of sample are shown in **Supplementary Table 1**.

All isolates were resistant to ticarcillin, ticarcillin/clavulanic acid, piperacillin, piperacillin/tazobactam and ciprofloxacin. High resistance rates were also found for both imipenem and meropenem (94.4%). Resistance to gentamicin was observed in 80.5% of the isolates, whereas 75% of the isolates were resistant to tobramycin and trimethoprim/sulfamethoxazole. It is worth mentioning that cefiderocol resistance was found in 22.2% of the isolates and 16.7% were resistant to minocycline. No colistin-resistant isolates were found (**Supplementary Table 2**).

### 3.2 Antibiotic resistance genes identification by Whole Genome Sequencing

Sequencing results showed 8 different *bla*_OXA-51-like_ variants including *bla*_OXA-51_, *bla*_OXA-64_, *bla*_OXA-65_, *bla*_OXA-66_, *bla*_OXA-68_, *bla*_OXA-91_, *bla*_OXA-94_ and *bla*_OXA-336_ (**Table 1**). Genes *bla*_OXA-66_ and *bla*_OXA-65_ were found in 16 and 10 isolates, respectively. Thirty-four isolates contained the *bla*_OXA-23_ gene, but the isolate Ale28 showed a 4bp deletion at position 203 of the gene leading to a premature stop codon at position 203 causing a lack of the protein expression. The presence of the *bla*_NDM-1_ carbapenemase gene was detected in ten isolates. Other β-lactamase genes such as *bla*_GES-35_ (6), *bla*_GES-11_ (3), *bla*_PER-7_ (4), *bla*_ADC-73_ (15), *bla*_ADC-117_ (9), *bla*_ADC-211_ (1), *bla*_ADC-143_ (2), *bla*_ADC-263_ (2), *bla*_ADC-80_ (3), *bla*_ADC-25_ (1), *bla*_ADC-259_ (1), *bla*_ADC-57_ (1), *bla*_ADC-52_ (1), *bla*_ADC-199_ (1) and *bla*_TEM-1_ (15) were detected.

**Table 1:** Clonal lineages (Pasteur Sequence Type (ST) and International Clone (IC)) and β-lactamase genes identified through sequencing experiments.

Isolate	International Clone	Pasteur ST	*bla*_OXA-51-like_	*bla*_OXA-23-like_	*bla*_NDM-like_	*bla*_GES-like_	*bla*_PER-like_	*bla*_ADC-like_	*bla*_TEM-like_
**Ale1**	IC2	600	*bla*_OXA-66_	*bla*_OXA-23_	*bla*_NDM-1_	–	–	*bla*_ADC-73_	*bla*_TEM-1_
**Ale2**	IC2	2	*bla*_OXA-66_	*bla*_OXA-23_	–	–	–	*bla*_ADC-73_	*bla*_TEM-1_
**Ale3**	IC2	570	*bla*_OXA-66_	*bla*_OXA-23_	–	–	–	*bla*_ADC-73_	*bla*_TEM-1_
**Ale4**	IC2	570	*bla*_OXA-66_	*bla*_OXA-23_	–	–	–	*bla*_ADC-73_	*bla*_TEM-1_
**Ale7**	IC2	570	*bla*_OXA-66_	*bla*_OXA-23_	*bla*_NDM-1_	–	–	*bla*_ADC-73_	*bla*_TEM-1_
**Ale8**	IC5	158	*bla*_OXA-65_	–	–	*bla*_GES-35_	–	*bla*_ADC-117_	–
**Ale9**	IC8	613	*bla*_OXA-68_	*bla*_OXA-23_	–	–	*bla*_PER-7_	*bla*_ADC-211_	–
**Ale10**	IC2	2	*bla*_OXA-66_	*bla*_OXA-23_	–	–	*bla*_PER-7_	*bla*_ADC-143_	–
**Ale11**	IC2	600	*bla*_OXA-66_	*bla*_OXA-23_	*bla*_NDM-1_	–	–	*bla*_ADC-73_	*bla*_TEM-1_
**Ale12**	IC5	158	*bla*_OXA-65_	–	–	*bla*_GES-35_	–	*bla*_ADC-73_	–
**Ale13**	IC2	570	*bla*_OXA-66_	*bla*_OXA-23_	*bla*_NDM-1_	–	–	*bla*_ADC-73_	*bla*_TEM-1_
**Ale14**	IC2	570	*bla*_OXA-336_	*bla*_OXA-23_	*bla*_NDM-1_	–	–	*bla*_ADC-73_	*bla*_TEM-1_
**Ale15**	IC4	15	*bla*_OXA-51_	*bla*_OXA-23_	–	–	–	*bla*_ADC-263_	–
**Ale16**	IC9	85	*bla*_OXA-94_	*bla*_OXA-23_	–	*bla*_GES-11_	–	*bla*_ADC-80_	–
**Ale17**	IC2	2	*bla*_OXA-66_	*bla*_OXA-23_	–	–	*bla*_PER-7_	*bla*_ADC-143_	–
**Ale18**	IC9	85	*bla*_OXA-94_	*bla*_OXA-23_	–	*bla*_GES-11_	–	*bla*_ADC-80_	–
**Ale19**	IC9	85	*bla*_OXA-94_	*bla*_OXA-23_	–	*bla*_GES-11_	–	*bla*_ADC-80_	–
**Ale20**	IC2	570	*bla*_OXA-66_	*bla*_OXA-23_	*bla*_NDM-1_	–	–	*bla*_ADC-25_	*bla*_TEM-1_
**Ale21**	IC4	15	*bla*_OXA-51_	*bla*_OXA-23_	–	–	–	*bla*_ADC-263_	–
**Ale22**	IC4	15	*bla*_OXA-51_	*bla*_OXA-23_	–	–	–	*bla*_ADC-259_	*bla*_TEM-1_
**Ale23**	IC5	158	*bla*_OXA-65_	*bla*_OXA-23_	–	–	–	*bla*_ADC-117_	–
**Ale24**	IC2	2	*bla*_OXA-66_	*bla*_OXA-23_	–	–	–	*bla*_ADC-73_	–
**Ale25**	IC7	113	*bla*_OXA-64_	*bla*_OXA-23_	*bla*_NDM-1_	–	*bla*_PER-7_	*bla*_ADC-57_	–
**Ale26**	IC5	158	*bla*_OXA-65_	*bla*_OXA-23_	–	*bla*_GES-35_	–	*bla*_ADC-117_	–
**Ale27**	IC2	2	*bla*_OXA-66_	*bla*_OXA-23_	–	–	–	*bla*_ADC-73_	*bla*_TEM-1_
**Ale28**	IC5	158	*bla*_OXA-65_	*bla*_OXA-23*_	–	–	–	*bla*_ADC-117_	–
**Ale29**	IC2	600	*bla*_OXA-66_	*bla*_OXA-23_	*bla*_NDM-1_	–	–	*bla*_ADC-73_	*bla*_TEM-1_
**Ale30**	Singleton	164	*bla*_OXA-91_	*bla*_OXA-23_	–	–	–	*bla*_ADC-52/ADC-199_	–
**Ale31**	IC5	158	*bla*_OXA-65_	*bla*_OXA-23_	–	–	–	*bla*_ADC-117_	–
**Ale32**	IC5	158	*bla*_OXA-65_	*bla*_OXA-23_	–	–	–	*bla*_ADC-117_	–
**Ale33**	IC5	158	*bla*_OXA-65_	*bla*_OXA-23_	–	*bla*_GES-35_	–	*bla*_ADC-117_	–
**Ale34**	IC5	158	*bla*_OXA-65_	*bla*_OXA-23_	–	*bla*_GES-35_	–	*bla*_ADC-117_	–
**Ale35**	IC2	600	*bla*_OXA-66_	*bla*_OXA-23_	*bla*_NDM-1_	–	–	*bla*_ADC-73_	*bla*_TEM-1_
**Ale36**	IC5	158	*bla*_OXA-65_	*bla*_OXA-23_	–	*bla*_GES-35_	–	*bla*_ADC-117_	–
**Ale37**	IC2	2	*bla*_OXA-66_	*bla*_OXA-23_	–	–	–	*bla*_ADC-73_	*bla*_TEM-1_
**Ale38**	IC2	570	*bla*_OXA-66_	*bla*_OXA-23_	*bla*_NDM-1_	–	–	*bla*_ADC-73_	*bla*_TEM-1_

*bla_OXA-23_ gene showed a deletion.

Other antibiotic resistance genes were also detected including genes conferring resistance to trimethoprim (*dfrA7)*, tetracyclines *(tet(B), tet(39))*, sulfonamides *(sul1, sul2)*, aminoglycosides *(armA, strA, strB, aph(3’)-Ia, aph(3’)-VI, aph(3’)-VIa, aac(6’)-Ib, ant(3’’)-II, ant(3’’)-IIa, aadA1-pm)*, macrolides *(mph(E), msr(E))*, rifamycin *(arr-2)* and chloramphenicol *(cmlA5, catB8, catA1)* (**Table 2**). Genes conferring resistance to aminoglycosides were especially abundant and present in the majority of the isolates. Genes coding for efflux pumps and their regulators were also detected including *abeM* (MATE family); *abeS* (SMR family); *amvA, abaF* and *abaQ* (MFS family); *adeA, adeB, adeC, adeF, adeG, adeH, adeI, adeJ,adeK, adeL, adeN, adeR* and *adeS* (RND family).

**Table 2:** Additional antibiotic resistance genes detected by Whole Genome Sequencing.

Isolates	Trimethoprim	Tetracyclines	Sulfonamides	Aminoglycosides	Macrolides	Rifamycin	Chloramphenicol
**Ale1**	*-*	*-*	*-*	*armA, aph(3´)-Ia, aph(3´)-VI, ant(3’’)-IIa, ant(3’’)-IIa*	*mph(E), msr(E)*	*-*	*-*
**Ale2**	*-*	*tet(B)*	*-*	*armA, strA, strB, aph(3´)-Ia, aph(3´)-VI, ant(3’’)-II*	*mph(E), msr(E)*	*-*	*-*
**Ale3**	*-*	*-*	*-*	*aph(3´)-Ia, ant(3’’)-II*	*mph(E), msr(E)*	*-*	*-*
**Ale4**	*-*	*-*	*-*	*aph(3´)-Ia, ant(3’’)-II*	*mph(E), msr(E)*	*-*	*-*
**Ale7**	*-*	*-*	*sul1*	*armA, aph(3´)-VI, aac(6’)-Ib, ant(3’’)-II, aadA1-pm*	*mph(E), msr(E)*	*-*	*catB8*
**Ale8**	*dfrA7*	*-*	*sul1*	*aph(3´)-VI, aac(6’)-Ib, ant(3’’)-II*	*-*	*-*	*-*
**Ale9**	*-*	*tet(B), tet(39)*	*sul1, sul2*	*armA, strA, strB, aph(3´)-VI, ant(3’’)-IIa*	*mph(E), msr(E)*	*arr-2*	*cmlA5*
**Ale10**	*-*	*-*	*sul1, sul2*	*armA, strA, strB, aph(3´)-Ia, aac(6’)-Ib, ant(3’’)-II, aadA1-pm*	*mph(E), msr(E)*	*-*	*catB8*
**Ale11**	*-*	*-*	*-*	*armA, aph(3´)-Ia, aph(3´)-VI, ant(3’’)-IIa*	*mph(E), msr(E)*	*-*	*-*
**Ale12**	*dfrA7*	*-*	*sul1*	*aph(3´)-VI, aac(6’)-Ib, ant(3’’)-II*	*-*	*-*	*-*
**Ale13**	*-*	*-*	*sul1*	*armA, aph(3´)-Ia, aac(6’)-Ib, ant(3’’)-II, aadA1-pm*	*mph(E), msr(E)*	*-*	*catB8*
**Ale14**	*-*	*-*	*sul1, sul2*	*armA, aph(3´)-VI, aac(6’)-Ib, ant(3’’)-II, aadA1-pm*	*mph(E), msr(E)*	*-*	*catB8*
**Ale15**	*-*	*-*	*sul1*	*armA, strA, strB, aph(3’)-Via, ant(3’’)-II*	*mph(E), msr(E)*	*arr-2*	*cmlA5*
**Ale16**	*dfrA7*	*-*	*sul1*	*aph(3´)-VI, aac(6’)-Ib, ant(3’’)-II*	*-*	*-*	*-*
**Ale17**	*-*	*-*	*sul1, sul2*	*armA, strA, strB, aph(3´)-Ia, aac(6’)-Ib, ant(3’’)-II, aadA1-pm*	*mph(E), msr(E)*	*-*	*catB8*
**Ale18**	*dfrA7*	*-*	*sul1*	*aph(3´)-VI, aac(6’)-Ib, ant(3’’)-II*	*-*	*-*	*-*
**Ale19**	*dfrA7*	*-*	*sul1*	*aph(3´)-VI, aac(6’)-Ib, ant(3’’)-II*	*-*	*-*	*-*
**Ale20**	*-*	*-*	*sul1*	*armA, aph(3´)-VI, aac(6’)-Ib, ant(3’’)-II, aadA1-pm*	*mph(E), msr(E)*	*-*	*catB8*
**Ale21**	*-*	*-*	*sul1*	*armA, strA, strB, aph(3’)-VIa, ant(3’’)-II*	*mph(E), msr(E)*	*arr-2*	*cmlA5*
**Ale22**	*-*	*-*	*sul1, sul2*	*armA, strA, strB, aph(3´)-VI, ant(3’’)-II*	*mph(E), msr(E)*	*arr-2*	*cmlA5*
**Ale23**	*-*	*-*	*sul1*	*armA, aph(3´)-Ia, aph(3´)-VI, aac(6’)-Ib, ant(3’’)-II, aadA1-pm*	*mph(E), msr(E)*	*-*	*catB8*
**Ale24**	*-*	*tet(B)*	*-*	*armA, strA, strB, aph(3´)-VI, ant(3’’)-II*	*mph(E), msr(E)*	*-*	*catA1*
**Ale25**	*-*	*tet(B)*	*sul1, sul2*	*armA, strA, strB, aph(3´)-VI, ant(3’’)-IIa*	*mph(E), msr(E)*	*arr-2*	*cmlA5*
**Ale26**	*-*	*-*	*sul1*	*armA, aph(3´)-Ia, aph(3´)-VI, ant(3’’)-II, aadA1-pm*	*mph(E), msr(E)*	*-*	*catB8*
**Ale27**	*-*	*tet(B)*	*-*	*armA, strA, strB, aph(3´)-Ia, aph(3´)-VI, ant(3’’)-II*	*mph(E), msr(E)*	*-*	*-*
**Ale28**	*dfrA7*	*-*	*sul1*	*armA, aph(3´)-Ia, aph(3´)-VI, aac(6’)-Ib, ant(3’’)-II, aadA1-pm*	*mph(E), msr(E)*	*-*	*catB8*
**Ale29**	*-*	*-*	*-*	*armA, aph(3´)-Ia, aph(3´)-VI, ant(3’’)-IIa*	*mph(E), msr(E)*	*-*	*-*
**Ale30**	*-*	*tet(39)*	*-*	*aph(3´)-VI, ant(3’’)-II*	*mph(E), msr(E)*	*-*	*-*
**Ale31**	*dfrA7*	*-*	*sul1*	*armA, aph(3´)-Ia, aph(3´)-VI, ant(3’’)-II, aadA1-pm*	*mph(E), msr(E)*	*-*	*catB8*
**Ale32**	*dfrA7*	*-*	*sul1*	*armA, aph(3´)-Ia, aph(3´)-VI, ant(3’’)-II, aadA1-pm*	*mph(E), msr(E)*	*-*	*catB8*
**Ale33**	*dfrA7*	*-*	*sul1*	*armA, aph(3´)-Ia, aph(3´)-VI, ant(3’’)-II, aadA1-pm*	*mph(E), msr(E)*	*-*	*catB8*
**Ale34**	*dfrA7*	*-*	*sul1*	*armA, aph(3´)-Ia, aph(3´)-VI, ant(3’’)-II, aadA1-pm*	*mph(E), msr(E)*	*-*	*catB8*
**Ale35**	*-*	*-*	*-*	*armA, aph(3´)-Ia, aph(3´)-VI, ant(3’’)-IIa*	*mph(E), msr(E)*	*-*	*-*
**Ale36**	*dfrA7*	*-*	*sul1*	*aph(3´)-VI, aac(6’)-Ib, ant(3’’)-II*	*-*	*-*	*-*
**Ale37**	*-*	*tet(B)*	*-*	*armA, strA, strB, aph(3´)-Ia, aph(3´)-VI, ant(3’’)-II*	*mph(E), msr(E)*	*-*	*-*
**Ale38**	*-*	*-*	*-*	*aph(3´)-Ia, aph(3´)-VI, ant(3’’)-II*	*-*	*-*	*-*

### 3.3 Genetic surroundings of β-lactamase genes

**Figure 1:**
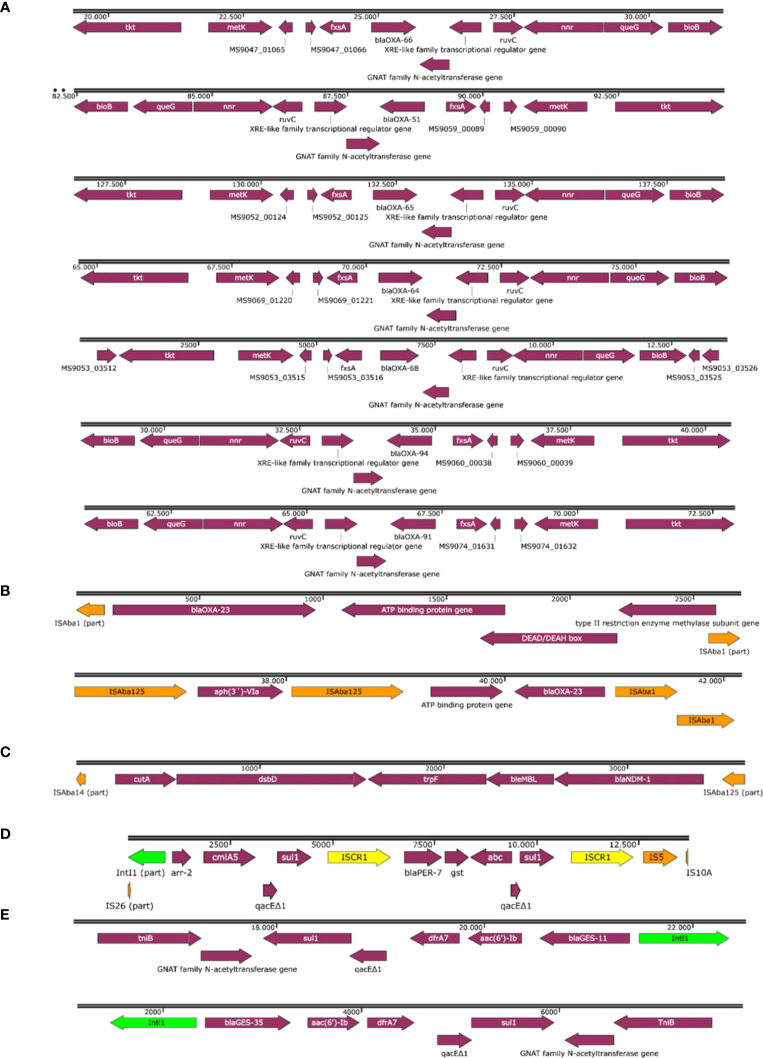
Genetic contexts of *bla*_OXA-51-like_ variants **(A)**, *bla*_OXA-23_ **(B)**, *bla*_NDM-1_ **(C)**, *bla*_PER-7_ **(D)** and *bla*_GES-like_ variants **(E)**.

The genetic contexts of the β-lactamase coding genes are shown in **Figure 1**. Regarding to *bla*_OXA-23_, it was located within Tn2006 in the singleton and isolates belonging to IC2, IC4 and IC7; and within Tn2008 transposons in isolates belonging to IC9 and IC5 (**Figure 1B**). In all the isolates harboring *bla*_NDM-1_, the gene was within the truncated isoform of transposon Tn125 (ΔTn125) (**Figure 1C**). We found the *bla*_PER-7_ gene located within a complex structure of IS*CR1* element and class 1 integron with part of IS26 upstream the integron in all the isolates carrying the gene (**Figure 1D**). The *bla*_GES-like_ genes were located within a class 1 integron accompanied by other resistance genes such as *aac(6´)-Ib, dfrA7* and *sul1* (**Figure 1E**). We did not find insertion sequences upstream any of the *bla*_OXA-51-like_ variants (**Figure 1A**).

### 3.4 Molecular typing and clonal relatedness

**Figure 2:**
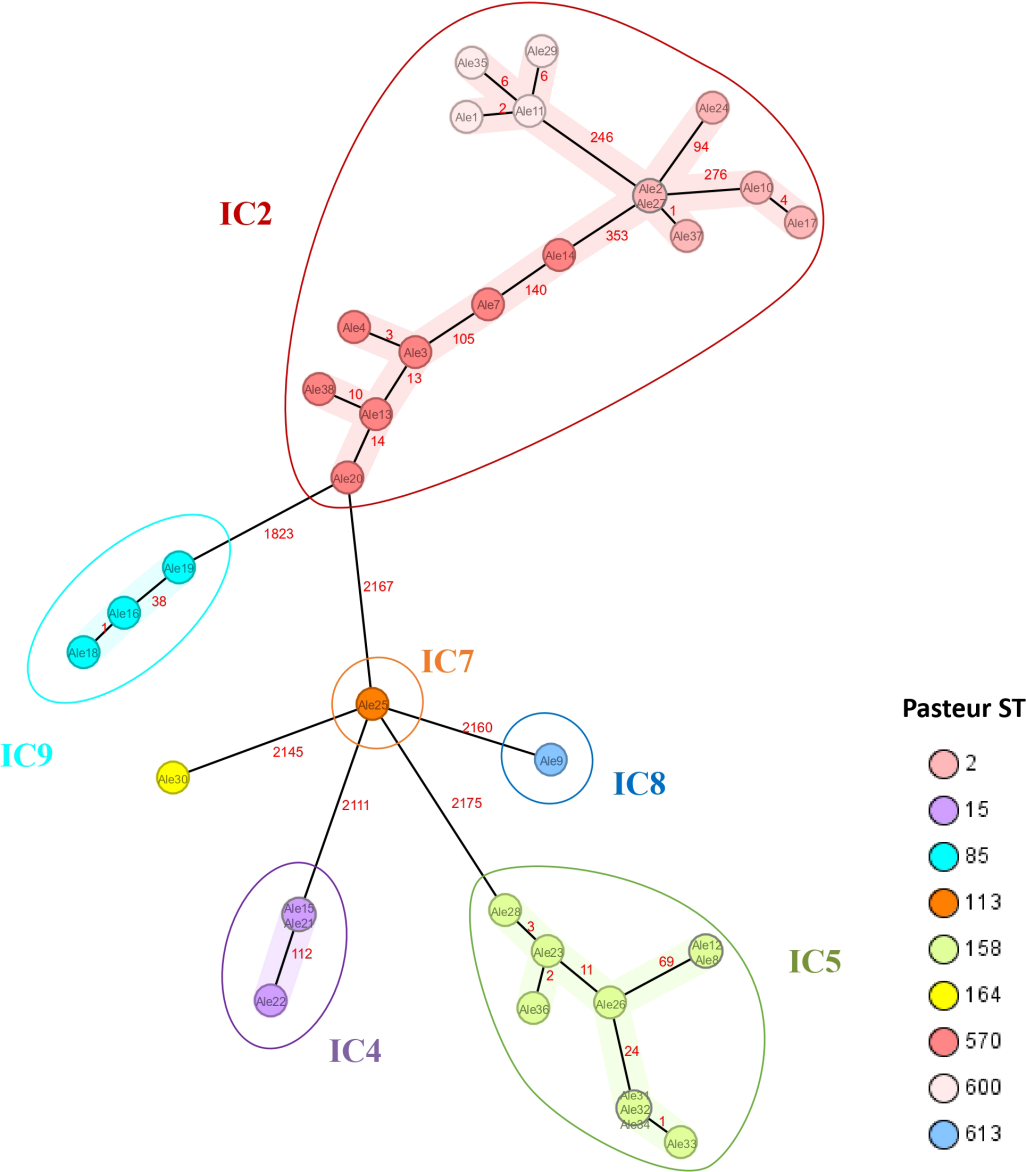
Minimum spanning tree of 36 A*. baumannii* isolates based on 2390 target alleles (core genome) generated using Ridom SeqSphere+ software. The study IDs of the isolates are shown within the nodes. Isolates are colored based on the assigned Pasteur STs and clustered by the International Clones they belong to.

Isolates were assigned to nine Pasteur STs and clustered in six different International Clones, including IC2, IC4, IC5, IC7, IC8 and IC9 (**Figure 2**). One isolate assigned to ST164 was not related to any described IC.

### 3.5 Virulence factors analysis

Virulome analysis of each International Clone and the singleton showed the presence of multiple virulence factors responsible for adherence (*Acinetobacter* trimeric autotransporter ATA and type IV pili), biofilm production (AdeFGH efflux pump, biofilm associated protein BAP, Csu fimbriae, Poly-N-acetyl-D-glucosamine, biofilm-controlling response regulator and quorum sensing), type II and type VI secretion systems, exotoxins (phospholipases C and D), exoenzymes (coagulation targeting metallo-endopeptidase CpaA), immune modulation (capsule, lipopolysaccharide, outer membrane protein OmpA and penicillin-binding Protein G) and iron uptake (acinetobactin and HemO cluster). The gene coding for the coagulation targeting metallo-endopeptidase (*cpaA*) was just observed in IC8 and IC9, and *ata* gene coding for *Acinetobacter* trimeric autotransporter was present in isolates from IC2, IC4, IC5 and in the singleton. The *bap* gene was detected in the isolates belonging to IC2, IC4, IC5 and IC8.

### 3.6 Motility phenotypes

Different motility phenotypes were observed in all isolates, except to Ale36 and Ale1. A cloud-like morphology with well-defined edges was observed in the majority of isolates, although some isolates radiated uniformly from the inoculation point presenting a positive control-like morphology.

### 3.7 Biofilm formation assays

**Figure 3:**
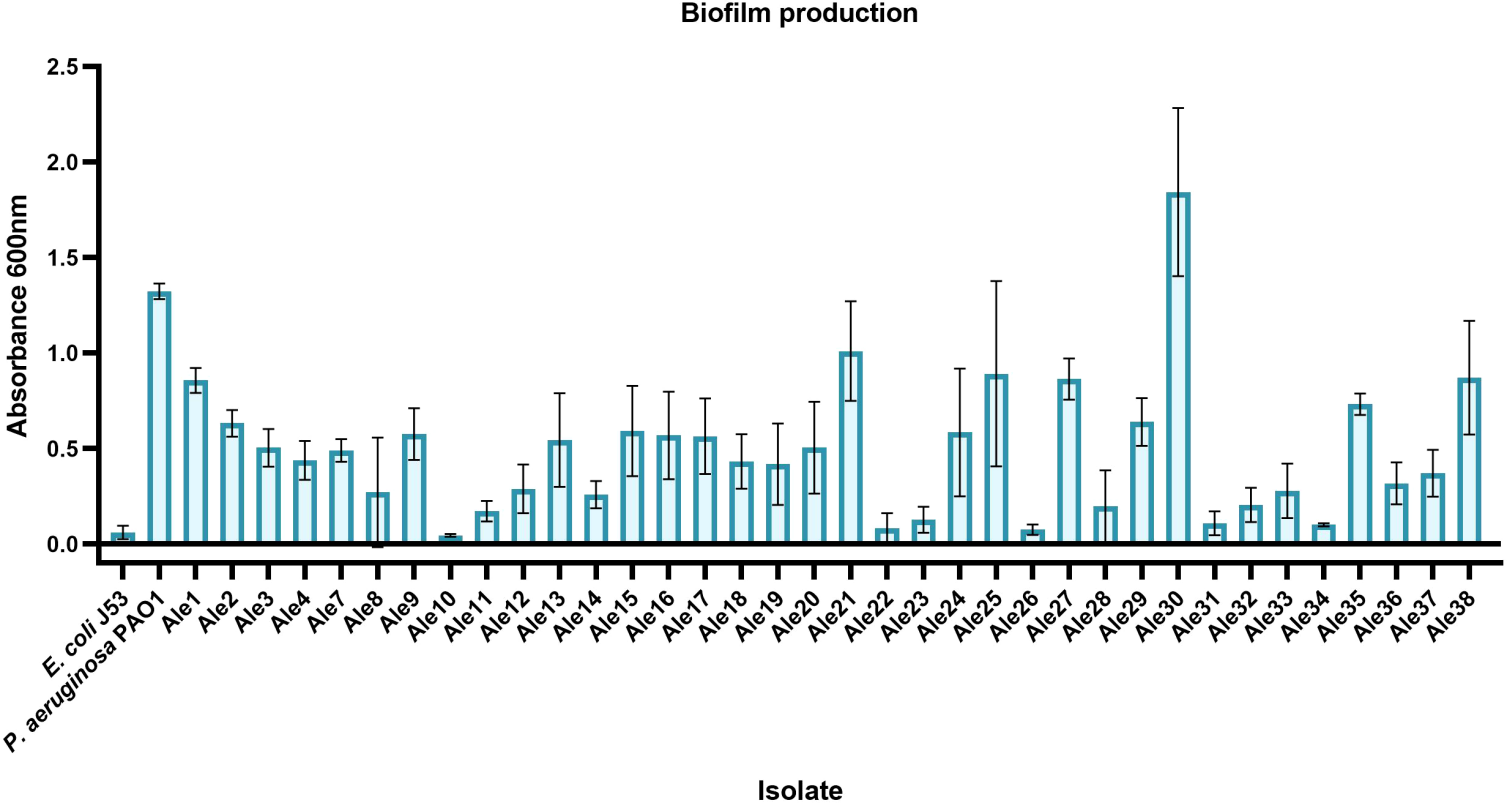
Biofilm production means ± standard deviations of the *A. baumannii* isolates. E. coli J53 and P. aeruginosa PAO1 were used as negative and positive controls, respectively.

Varying degrees of biofilm production were observed among the isolates (**Figure 3**). It was observed that stronger biofilm-producers belonged to IC2, IC4 and IC7 and were isolated from BAL/miniBAL and swabs (Ale30, Ale21, Ale25, Ale38, Ale27 and Ale1). Especially remarkable was the strong biofilm production capacity of the singleton Ale30, which showed even a higher biofilm production than the positive control.

### 3.8 Plasmid analysis and genetic localization of *bla*_NDM-1_, *bla*_PER-7_ and *bla*_GES-like_ genes

**Figure 4:**
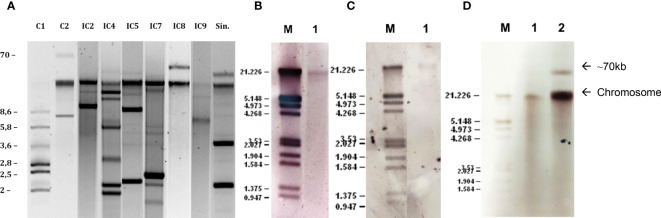
Plasmid profiles and hybridization experiments of the *A. baumannii* isolates. **(A)** plasmid profiles of the six identified International Clones and the singleton (Sin.); **(B)** hybridization signal in the chromosome with the *bla*_NDM-1_ probe; **(C)** hybridization signal in the chromosome with the *bla*_PER-7_ probe; **(D)** hybridization signal in the chromosome with the *bla*_GES-like_ probe in lane 1 and hybridization signal in a 70kb plasmid in lane 2.

Plasmids ranging in size from 1.7 to 70 kb were observed (**Figure 4A**). Hybridization experiments located the *bla*_NDM-1_ gene in the chromosome (**Figure 4B**). Chromosomic localization of *bla*_PER-7_ gene was also confirmed (**Figure 4C**). The *bla*_GES-like_ genes were located in the chromosome in all the isolates, and simultaneously located in a plasmid of approximately 70kb in five isolates (**Figure 4D**). Replicon typing experiments showed the presence of genes coding for previously described replicases Aci1/Aci2, Aci4, Aci6, Aci8/Aci9, p2S1 and pAB49 pertaining to homology groups 2, 4, 6, 8, 12 and 16, respectively, in all the isolates except to Ale4. Twenty-one isolates showed a combination of two replicases: Aci1/Aci2 + Aci6 (6); Aci1/Aci2 + p2S1 (1); p2S1+Aci6 (10); Aci8/9 + Aci6 (1); Aci4+Aci8/Aci9 (3).

## 4 Discussion

It is said that the lack of regulation and the abuse in the use of antibiotics in Egypt are the main cause of the acceleration in the emergence of resistant isolates, and also responsible for exporting resistance to other countries (Elwakil et al., 2023). In the present study, 94.4% of carbapenem, 100% of fluoroquinolone and 86.11% of aminoglycoside resistance was observed, which is consistent with previous studies reporting a carbapenem resistance of 98% with elevated levels of resistance to quinolones and aminoglycosides in Mansoura, Egypt (Said et al., 2018). In fact, our isolates showed higher aminoglycoside resistance ratios than the resistance reported in recent studies from Egypt (up to 82% and 67%) (ELsheredy et al., 2021; Kishk et al., 2021). To our knowledge, up to date, no cefiderocol resistant isolates have been reported in Egypt apart from the eight resistant isolates analyzed in our study. Fortunately, isolates remained susceptible to colistin, although colistin resistance was reported in up to 53% of *A. baumannii* Egyptian isolates according to studies published in 2020 (Fam et al., 2020; Makharita et al., 2020).

WGS analysis showed *bla*_OXA-66_ and *bla*_OXA-65_ as the most frequent *bla*_OXA-51-like_ variants among the isolates, which is consistent with the high prevalence of *bla*_OXA-66_ in North Africa and the Middle East (Muthuirulandi Sethuvel et al., 2019) but not with the reported prevalence of *bla*_OXA-65_ in Egypt (Elwakil et al., 2023). A higher prevalence of *bla*_OXA-51_ has been reported in Egypt during the last years, while other variants described in this study have been reported less frequently in Egypt (Elwakil et al., 2023). The genetic contexts of the *bla*_OXA-51-like_ variants found in our isolates, were similar to those found in previously described isolates (Vijayakumar et al., 2022). Although IS*Aba* is a commonly found element upstream these genes, not all the variants harbor it necessarily. The most common acquired carbapenemase gene found in these isolates was *bla*_OXA-23_, which is usually found in 90–100% of carbapenem-resistant *A. baumannii* isolates in Egypt (Abouelfetouh et al., 2019; Hassan et al., 2021). This gene was located within Tn2006 or Tn2008 transposons as previously described, which is crucial for the overexpression and mobilization of *bla*_OXA-23_ (Hamidian and Nigro, 2019). Regarding to *bla*_GES-like_ genes, these type of Extended-Spectrum β-Lactamase genes have been increasingly reported during the last years, and some of its variants can possess carbapenemase activity (Bonnin Rémy et al., 2013). These type of β-Lactamase genes are frequently reported in the Mediterranean Area and the Middle East countries such as Turkey, Tunisia, or Kuwait (Bonnin Rémy et al., 2013; Cicek et al., 2014; Chihi et al., 2016). However, in our study, these genes were identified in 25% of the isolates, half of the frequency reported in other studies in Egypt (50%) (Ramadan et al., 2018). As previously described in isolates from Pakistan, *bla*_GES-like_ genes were part of a class 1 integron co-harboring *dfrA7, sul1* and *aac(6’)-Ib* resistance genes (Karah et al., 2020). Furthermore, *bla*_OXA-23_ and *bla*_GES-11_ genes have been described encoded in a large conjugative plasmid named pK50a (79.6kb) which is a member of the Aci6 group (Wibberg et al., 2018). This is approximately of the same size as the plasmid in which we identified the *bla*_GES-11_ gene, and it is worth mentioning that all the isolates harboring *bla*_GES-like_ genes were positive for replicase Aci6 too. The *bla*_NDM-1_ gene was detected in 27.8% of isolates, in concordance with the literature where NDM-type carbapenemases are commonly reported in Egyptian isolates with a prevalence of 0–39.3% (El-Kholy et al., 2021). This gene was found within the truncated isoform of Tn125 (ΔTn125) in our isolates with the characteristic IS*Aba125* upstream of the *bla*_NDM-1_ gene which appears to enhance its expression (Wang et al., 2012). In all the isolates, *bla*_NDM-1_ gene was chromosome-borne, which is the most frequent localization (Fernández-Cuenca et al., 2020). The *bla*_PER-7_ gene was located within a complex structure connecting IS*CR1* element and class 1 integron, which is a previously described structure closely related to multidrug resistant bacteria (Cheng et al., 2016). However, another IS*CR1* element, IS5 and part of IS10A were identified downstream of the 3’-CS which differs from the described structure. NDM-type carbapenemases and PER-type β-lactamases seem to be involved in cefiderocol resistance, the recently developed last resort antibiotic (Naeimi Mazraeh et al., 2021; Poirel et al., 2021).

Analysis of the sequenced genomes showed that seventeen isolates belonged to IC2 with the characteristic *bla*_OXA-66_ gene, which is the dominant clone circulating worldwide and in Egypt (Hassan et al., 2021). Among this lineage, ST570 was the most abundant clade, an ST which has four entries in the PubMLST database, submitted from Vietnam (1) and Egypt (3) indicating that this lineage is circulating in Egyptian health settings at least, since 2017. Regarding to ST2, among 1319 isolates submitted to the database, two entries from Egypt between 2013 and 2015, and eight entries from Jordan between 2019 and 2020 were found. A recent study showed six out of seven Syrian strains pertaining to ST2 (Higgins et al., 2021), demonstrating that ST2 is commonly circulating between countries of the Mediterranean area. The less abundant clade was ST600, which has five entries in the PubMLST database isolated from Jordan (4) and Libya (1), between 2014 and 2020, suggesting a possible transmission between these two nearby countries during these years. Ten isolates belonged to IC5 (ST158) harbouring the characteristic *bla*_OXA-65_ gene. Four isolates were identified in the PubMLST database isolated from Iraq, Turkey, Russia and Egypt; consistent with a study published in 2020 in Egypt, where the majority of the isolates belonged to ST158, indicating that this clone is circulating in the Mediterranean and Middle East area (Elwakil et al., 2023). Three isolates belonged to IC4 (ST15), a ST which is predominantly found in Latin American countries, although it has also been described in countries of the Mediterranean are such as Turkey (Di Popolo et al., 2011). Three isolates were related to IC9 (ST85), which have been recently reported in Libya (Higgins et al., 2021). A search in PubMLST also showed four entries in Jordan during 2020 and one in Egypt in 2017 demonstrating the presence of this clade in Egypt and the Middle East area. Just one isolate belonging to IC7(ST113) was identified, a sequence type frequently reported in South America (Kurihara et al., 2020) although, it has also been described in Cairo in 5 isolates from 2018 to 2020 according to PubMLST. A single isolate pertaining to IC8(ST613) was detected, this is a linage with little presence in the Middle East and North Africa, with just one isolate reported in Alexandria in 2013 according to PubMLST. A singleton assigned to ST164 was identified among our isolates, this ST have been identified in Germany in 2021 (Wareth et al., 2021) and in Turkey in 2016. There are no public records of isolates belonging to this linage in Egypt up to date.

In conclusion, this study showed a high clonal diversity among CRAB isolates collected from hospitals in Alexandria, and highlights the emergence of not frequently reported lineages in Egypt. The high incidence of *bla*_OXA-23_ carbapenemase as well as *bla*_NDM-1_ is of concern as they are key in carbapenem resistance and to many other antibiotics. This work also puts the spotlight in the emergence of cefiderocol resistant isolates in Egyptian hospitals. It becomes necessary to harden infection control measures and to increase epidemiologic studies in Egypt to limit the development of new clones with highly resistant genes.

## Data availability statement

The datasets presented in this study can be found in online repositories. The names of the repository/repositories and accession number(s) can be found in the article/**Supplementary Material**.

## Author contributions

SS-U, LG and IA contributed to the design of the experiments and were responsible for the project funding. SS-U, AM-B, AO-S, MH and DA performed the experiments. SS-U, AO-S and LG analyzed and interpreted the data. SS-U and LG wrote the manuscript. SMS and ME-K were responsible for the collection, identification and determination of the resistance profile of the isolates. All authors contributed to the article and approved the submitted version.
