# Supplementary material for: Molecular characterization of multidrug resistant Acinetobacter baumannii clinical isolates from Alexandria, Egypt
Source: Front Cell Infect Microbiol. 2023 Jul 20;13:1208046. doi: 10.3389/fcimb.2023.1208046 (PMC10399577; doi:10.3389/fcimb.2023.1208046)
Supplement: Supplementary file 1 [file DataSheet_1.docx]

Supplementary Material

Molecular characterization of multidrug resistant *Acinetobacter baumannii* clinical isolates from Alexandria, Egypt

Sánchez-Urtaza^1^, Alain Ocampo-Sosa^2,3^, Ainhoa Molins-Bengoetxea^1^, Mohammed A. El-Kholy^4^, Marta Hernandez^5^, David Abad^5^, Sherine M. Shawky^6^, Itziar Alkorta^7^ and Lucia Gallego^1*^

*** Correspondence:** Lucia Gallego: [lucia.gallego@ehu.es](mailto:lucia.gallego@ehu.es)

# Supplementary Data

# Supplementary Figures and Tables

## Supplementary Figures


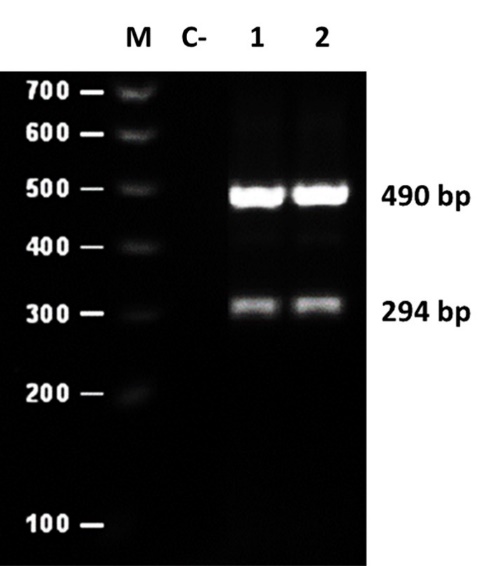


Supplementary Figure 1. Electrophoresis gel of *gyrB* multiplex PCR. Lane 1: Molecular weight marker ranging in size from 100 to 700 bp, lane 2: negative control, lane 3: *A. baumannii* ATCC 19606 and lane 4: *A. baumannii* Ale25.

## Supplementary Tables

Supplementary Table 1. Characteristics of the isolates recovered in the present study. BAL= Bronchoalveolar lavage.

| Isolate | Species | Collection date | Patient sex | Type of culture | Genbank Accession number |
| --- | --- | --- | --- | --- | --- |
| Ale1 | *A. baumannii* | 24/08/2020 | Male | BAL | JARNMD000000000 |
| Ale2 | *A. baumannii* | 20/08/2020 | Male | Swab | JARNMC000000000 |
| Ale3 | *A. baumannii* | 25/08/2020 | Female | MiniBAL | JARNMB000000000 |
| Ale4 | *A. baumannii* | 27/08/2020 | Male | MiniBAL | JARNMA000000000 |
| Ale7 | *A. baumannii* | 04/10/2020 | Male | Endotracheal tube | JARNLZ000000000 |
| Ale8 | *A. baumannii* | 06/10/2020 | Male | Swab | JARNLY000000000 |
| Ale9 | *A. baumannii* | 08/10/2020 | Female | MiniBAL | JARNLX000000000 |
| Ale10 | *A. baumannii* | 09/10/2020 | Female | Aspirate | JARNLW000000000 |
| Ale11 | *A. baumannii* | 10/10/2020 | Female | Swab | JARNLV000000000 |
| Ale12 | *A. baumannii* | 08/10/2020 | Male | Blood | JARNLU000000000 |
| Ale13 | *A. baumannii* | 14/10/2020 | Male | Aspirate | JARNLT000000000 |
| Ale14 | *A. baumannii* | 15/10/2020 | Male | MiniBAL | JARNLS000000000 |
| Ale15 | *A. baumannii* | 28/10/2020 | Female | MiniBAL | JARNLR000000000 |
| Ale16 | *A. baumannii* | 10/11/2020 | Male | MiniBAL | JARNLQ000000000 |
| Ale17 | *A. baumannii* | 09/11/2020 | Male | MiniBAL | JARNLP000000000 |
| Ale18 | *A. baumannii* | 05/11/2020 | Female | Urine | JARNLO000000000 |
| Ale19 | *A. baumannii* | 10/11/2020 | Male | Aspirate | JARNLN000000000 |
| Ale20 | *A. baumannii* | 12/11/2020 | Male | Sputum | JARNLM000000000 |
| Ale21 | *A. baumannii* | 12/11/2020 | Female | Swab | JARNLL000000000 |
| Ale22 | *A. baumannii* | 21/06/2020 | Female | Blood | JARNLK000000000 |
| Ale23 | *A. baumannii* | 22/11/2020 | Male | Tissue | JARNLJ000000000 |
| Ale24 | *A. baumannii* | 28/11/2020 | Female | Swab | JARNLI000000000 |
| Ale25 | *A. baumannii* | 29/11/2020 | Female | Swab | JANBZS000000000 |
| Ale26 | *A. baumannii* | 01/12/2020 | Female | MiniBAL | JARNLH000000000 |
| Ale27 | *A. baumannii* | 06/12/2020 | Female | Swab | JARNLG000000000 |
| Ale28 | *A. baumannii* | 07/12/2020 | Male | MiniBAL | JARNLF000000000 |
| Ale29 | *A. baumannii* | 11/12/2020 | Male | Sputum | JARNLE000000000 |
| Ale30 | *A. baumannii* | 11/12/2020 | Male | MiniBAL | JARNLD000000000 |
| Ale31 | *A. baumannii* | 25/12/2020 | Male | MiniBAL | JARNLC000000000 |
| Ale32 | *A. baumannii* | 28/12/2020 | Male | MiniBAL | JARNLB000000000 |
| Ale33 | *A. baumannii* | 01/01/2021 | Female | Swab | JARNLA000000000 |
| Ale34 | *A. baumannii* | 02/01/2021 | Male | Blood | JARNKZ000000000 |
| Ale35 | *A. baumannii* | - | Female | Blood | JARNKY000000000 |
| Ale36 | *A. baumannii* | 28/01/2021 | Male | Blood | JARNKX000000000 |
| Ale37 | *A. baumannii* | 07/02/2021 | Female | Sputum | JARNKW000000000 |
| Ale38 | *A. baumannii* | 08/02/2021 | Male | BAL | JARNKV000000000 |

Supplementary Table 2. Minimum Inhibitory Concentrations of the *A. baumannii* isolates obtained by VITEK2® automated system expressed in mg/L. Resistant and intermediate MIC values are represented in bold. Antibiotic abbreviations correspond to: ticarcillin (TIC), ticarcillin/clavulanic acid (TCC), piperacillin (PRL), piperacillin/tazobactam (TZP), imipenem (IPM), meropenem (MEM), gentamicin (GEN), tobramycin (TOB), ciprofloxacin (CIP), minocycline (MIN), colistin (CST), trimethoprim/sulfamethoxazole (SXT) and cefiderocol (FDC).

| MIC (mg/L) | | | | | | | | | | | | | |
| --- | --- | --- | --- | --- | --- | --- | --- | --- | --- | --- | --- | --- | --- |
| Isolate | TIC | TCC | PRL | TZP | IPM | MEM | GEN | TOB | CIP | MIN | CST | SXT | FDC |
| Ale1 | **≥ 128** | **≥ 128** | **≥ 128** | **≥ 128** | **≥ 16** | **≥ 16** | **≥ 16** | **≥ 16** | **≥ 4** | 4 | ≤ 0.5 | ≤ 20 | ≤ 2 |
| Ale2 | **≥ 128** | **≥ 128** | **≥ 128** | **≥ 128** | **≥ 16** | **≥ 16** | **8** | 4 | **≥ 4** | **≥ 16** | ≤ 0.5 | ≤ 20 | ≤ 2 |
| Ale3 | **≥ 128** | **≥ 128** | **≥ 128** | **≥ 128** | **≥ 16** | **≥ 16** | **≥ 16** | **≥ 16** | **≥ 4** | 2 | ≤ 0.5 | **160** | ≤ 2 |
| Ale4 | **≥ 128** | **≥ 128** | **≥ 128** | **≥ 128** | **8** | **≥ 16** | **≥ 16** | **≥ 16** | **≥ 4** | ≤ 1 | ≤ 0.5 | **≥ 320** | ≤ 2 |
| Ale7 | **≥ 128** | **≥ 128** | **≥ 128** | **≥ 128** | **≥ 16** | **≥ 16** | **≥ 16** | **≥ 16** | **≥ 4** | 2 | ≤ 0.5 | **≥ 320** | ≤ 2 |
| Ale8 | **≥ 128** | **≥ 128** | **≥ 128** | **≥ 128** | 1 | 4 | ≤ 1 | 2 | **≥ 4** | ≤ 1 | ≤ 0.5 | **≥ 320** | ≤ 2 |
| Ale9 | **≥ 128** | **≥ 128** | **≥ 128** | **≥ 128** | **≥ 16** | **≥ 16** | **≥ 16** | **≥ 16** | **≥ 4** | 4 | ≤ 0.5 | **≥ 320** | **> 2** |
| Ale10 | **≥ 128** | **≥ 128** | **≥ 128** | **≥ 128** | **≥ 16** | **≥ 16** | **≥ 16** | **≥ 16** | **≥ 4** | 2 | ≤ 0.5 | **≥ 320** | ≤ 2 |
| Ale11 | **≥ 128** | **≥ 128** | **≥ 128** | **≥ 128** | **≥ 16** | **≥ 16** | **≥ 16** | **≥ 16** | **≥ 4** | 4 | ≤ 0.5 | ≤ 20 | ≤ 2 |
| Ale12 | **≥ 128** | **≥ 128** | **≥ 128** | **≥ 128** | 1 | 2 | **≥ 16** | **≥ 16** | **≥ 4** | ≤ 1 | ≤ 0.5 | **≥ 320** | ≤ 2 |
| Ale13 | **≥ 128** | **≥ 128** | **≥ 128** | **≥ 128** | **≥ 16** | **≥ 16** | **≥ 16** | **≥ 16** | **≥ 4** | 4 | ≤ 0.5 | **160** | **> 2** |
| Ale14 | **≥ 128** | **≥ 128** | **≥ 128** | **≥ 128** | **≥ 16** | **≥ 16** | **≥ 16** | **≥ 16** | **≥ 4** | 4 | ≤ 0.5 | **≥ 320** | ≤ 2 |
| Ale15 | **≥ 128** | **≥ 128** | **≥ 128** | **≥ 128** | **≥ 16** | **≥ 16** | 4 | **≥ 16** | **≥ 4** | ≤ 1 | ≤ 0.5 | **160** | ≤ 2 |
| Ale16 | **≥ 128** | **≥ 128** | **≥ 128** | **≥ 128** | **≥ 16** | **≥ 16** | **≥ 16** | **≥ 16** | **≥ 4** | 2 | ≤ 0.5 | **≥ 320** | **> 2** |
| Ale17 | **≥ 128** | **≥ 128** | **≥ 128** | **≥ 128** | **≥ 16** | **≥ 16** | **≥ 16** | **≥ 16** | **≥ 4** | 2 | ≤ 0.5 | **≥ 320** | **> 2** |
| Ale18 | - | - | - | - | **≥ 16** | **≥ 16** | ≤ 1 | 4 | **≥ 4** | - | - | **≥ 320** | ≤ 2 |
| Ale19 | **≥ 128** | **≥ 128** | **≥ 128** | **≥ 128** | **≥ 16** | **≥ 16** | ≤ 1 | 2 | **≥ 4** | ≤ 1 | ≤ 0.5 | **≥ 320** | ≤ 2 |
| Ale20 | **≥ 128** | **≥ 128** | **≥ 128** | **≥ 128** | **≥ 16** | **≥ 16** | **≥ 16** | **≥ 16** | **≥ 4** | **8** | ≤ 0.5 | **160** | **> 2** |
| Ale21 | **≥ 128** | **≥ 128** | **≥ 128** | **≥ 128** | **≥ 16** | **≥ 16** | 4 | **≥ 16** | **≥ 4** | ≤ 1 | ≤ 0.5 | **160** | ≤ 2 |
| Ale22 | **≥ 128** | **≥ 128** | **≥ 128** | **≥ 128** | **≥ 16** | **≥ 16** | **8** | 2 | **≥ 4** | ≤ 1 | ≤ 0.5 | **≥ 320** | ≤ 2 |
| Ale23 | **≥ 128** | **≥ 128** | **≥ 128** | **≥ 128** | **≥ 16** | **≥ 16** | **≥ 16** | **≥ 16** | **≥ 4** | ≤ 1 | ≤ 0.5 | **160** | ≤ 2 |
| Ale24 | **≥ 128** | **≥ 128** | **≥ 128** | **≥ 128** | **≥ 16** | **≥ 16** | **≥ 16** | 4 | **≥ 4** | **≥ 16** | ≤ 0.5 | ≤ 20 | ≤ 2 |
| Ale25 | **≥ 128** | **≥ 128** | **≥ 128** | **≥ 128** | **≥ 16** | **≥ 16** | ≤ 1 | ≤ 1 | **≥ 4** | ≤ 1 | ≤ 0.5 | **≥ 320** | **> 2** |
| Ale26 | **≥ 128** | **≥ 128** | **≥ 128** | **≥ 128** | **≥ 16** | **≥ 16** | **≥ 16** | **≥ 16** | **≥ 4** | ≤ 1 | ≤ 0.5 | **≥ 320** | ≤ 2 |
| Ale27 | **≥ 128** | **≥ 128** | **≥ 128** | **≥ 128** | **≥ 16** | **≥ 16** | **≥ 16** | **8** | **≥ 4** | **≥ 16** | ≤ 0.5 | ≤ 20 | ≤ 2 |
| Ale28 | **≥ 128** | **≥ 128** | **≥ 128** | **≥ 128** | **≥ 16** | **≥ 16** | **≥ 16** | **≥ 16** | **≥ 4** | ≤ 1 | ≤ 0.5 | **≥ 320** | ≤ 2 |
| Ale29 | **≥ 128** | **≥ 128** | **≥ 128** | **≥ 128** | **≥ 16** | **≥ 16** | **≥ 16** | **≥ 16** | **≥ 4** | 2 | ≤ 0.5 | ≤ 20 | ≤ 2 |
| Ale30 | **≥ 128** | **≥ 128** | **≥ 128** | **≥ 128** | **≥ 16** | **≥ 16** | 4 | ≤ 1 | **≥ 4** | ≤ 1 | ≤ 0.5 | ≤ 20 | ≤ 2 |
| Ale31 | **≥ 128** | **≥ 128** | **≥ 128** | **≥ 128** | **≥ 16** | **≥ 16** | **≥ 16** | **≥ 16** | **≥ 4** | ≤ 1 | 1 | **≥ 320** | ≤ 2 |
| Ale32 | **≥ 128** | **≥ 128** | **≥ 128** | **≥ 128** | **≥ 16** | **≥ 16** | **8** | **≥ 16** | **≥ 4** | 2 | ≤ 0.5 | **≥ 320** | ≤ 2 |
| Ale33 | **≥ 128** | **≥ 128** | **≥ 128** | **≥ 128** | **≥ 16** | **≥ 16** | **≥ 16** | **≥ 16** | **≥ 4** | 4 | ≤ 0.5 | ≤ 20 | ≤ 2 |
| Ale34 | **≥ 128** | **≥ 128** | **≥ 128** | **≥ 128** | **≥ 16** | **≥ 16** | **≥ 16** | 4 | **≥ 4** | 2 | ≤ 0.5 | **≥ 320** | ≤ 2 |
| Ale35 | **≥ 128** | **≥ 128** | **≥ 128** | **≥ 128** | **≥ 16** | **≥ 16** | **≥ 16** | **≥ 16** | **≥ 4** | ≤ 1 | ≤ 0.5 | **≥ 320** | **> 2** |
| Ale36 | **≥ 128** | **≥ 128** | **≥ 128** | **≥ 128** | **≥ 16** | **≥ 16** | **≥ 16** | **≥ 16** | **≥ 4** | **≥ 16** | ≤ 0.5 | ≤ 20 | ≤ 2 |
| Ale37 | **≥ 128** | **≥ 128** | **≥ 128** | **≥ 128** | **≥ 16** | **≥ 16** | **≥ 16** | **≥ 16** | **≥ 4** | **≥ 16** | ≤ 0.5 | ≤ 20 | ≤ 2 |
| Ale38 | **≥ 128** | **≥ 128** | **≥ 128** | **≥ 128** | **≥ 16** | **≥ 16** | **≥ 16** | **≥ 16** | **≥ 4** | ≤ 1 | ≤ 0.5 | **80** | **> 2** |
